# Lipoprotein metabolism in patients with type 1 diabetes under intensive insulin treatment

**DOI:** 10.1186/1476-511X-12-15

**Published:** 2013-02-11

**Authors:** Alina C R Feitosa, Gilson S Feitosa-Filho, Fatima R Freitas, Bernardo L Wajchenberg, Raul C Maranhão

**Affiliations:** 1 Heart Institute (InCor) of the Medical School Hospital, University of Sao Paulo, São Paulo, Brazil; 2 Endocrinology Service of the Medical School Hospital, University of Sao Paulo, São Paulo, Brazil; 3 Faculty of Pharmaceutic Sciences, University of Sao Paulo, São Paulo, Brazil

**Keywords:** Type 1 diabetes mellitus, Insulin treatment, Glycemia, Lipoprotein, Cholesterol metabolism, Lipids transfer, Cardiovascular disease

## Abstract

**Background:**

Type 1 diabetes (T1DM) is frequently accompanied by dyslipidemia related with insulin-dependent steps of the intravascular lipoprotein metabolism. T1DM dyslipidemia may predispose to precocious cardiovascular disease and the lipid status in T1DM under intensive insulin treatment has not been sufficiently explored. The aim was to investigate the plasma lipids and the metabolism of LDL and HDL in insulin-treated T1DM patients with high glycemic levels.

**Methods:**

Sixteen male patients with T1DM (26 ± 7 yrs) with glycated hemoglobin >7%, and 15 control subjects (28 ± 6 yrs) were injected with a lipid nanoemulsion (LDE) resembling LDL and labeled with ^14^C-cholesteryl ester and ^3^H-free-cholesterol for determination of fractional clearance rates (FCR, in h^-1^) and cholesterol esterification kinetics. Transfer of labeled lipids from LDE to HDL was assayed in vitro.

**Results:**

LDL-cholesterol (83 ± 15 vs 100 ± 29 mg/dl, p=0.08) tended to be lower in T1DM than in controls; HDL-cholesterol and triglycerides were equal. LDE marker ^14^C-cholesteryl ester was removed faster from plasma in T1DM patients than in controls (FCR=0.059 ± 0.022 vs 0.039 ± 0.022h-1, p=0.019), which may account for their lower LDL-cholesterol levels. Cholesterol esterification kinetics and transfer of non-esterified and esterified cholesterol, phospholipids and triglycerides from LDE to HDL were also equal.

**Conclusion:**

T1DM patients under intensive insulin treatment but with poor glycemic control had lower LDL-cholesterol with higher LDE plasma clearance, indicating that LDL plasma removal was even more efficient than in controls. Furthermore, HDL-cholesterol and triglycerides, cholesterol esterification and transfer of lipids to HDL, an important step in reverse cholesterol transport, were all normal. Coexistence of high glycemia levels with normal intravascular lipid metabolism may be related to differences in exogenous insulin bioavailabity and different insulin mechanisms of action on glucose and lipids. Those findings may have important implications for prevention of macrovascular disease by intensive insulin treatment.

## Background

Patients with type 1 diabetes mellitus (T1DM) may present disturbances of plasma lipids such as hypertriglyceridemia and low HDL cholesterol and, less frequently, high LDL cholesterol [1]. T1DM patients also show defects in the regulation of the plasma lipid metabolism that are not routinely evaluated, such as increase in the small dense LDL subfraction [2], which is more atherogenic, and dysfunctional HDL. In addition, alterations in the transfer of lipids were found in those patients [3,4]. This process, in which lipids are exchanged among lipoprotein classes, depends on transfer proteins such as cholesteryl ester transfer protein (CETP) and phospholipid transfer protein (PLTP). Effective insulin treatment not only improves glycemia but also the plasma lipid profile.

Lipoprotein metabolism in the intravascular compartment can be probed by using artificial nanoemulsions (LDE) with lipid structure that resembles LDL [5]. This approach greatly facilitates the investigation of LDL metabolism, since with a single preparation labeled with radioactivity or other means can be tested in several patients. LDE is made without protein, but in contact with plasma, the nanoemulsion particles gain exchangeable apolipoproteins (apo), free or associated with the native lipoproteins. Apo E acquired by LDE allows binding of the LDE particles to LDL receptors and cell uptake via the receptor-mediated endocytic pathway. LDE was validated as a probe to detect changes in LDL metabolism in disease states or under the action of drugs or dietary interventions [6-8]. As an example, when injected into patients with familial hypercholesterolemia, wherein LDL receptors are defective, LDE was slowly removed from the circulation, as it occurs when native LDL is injected [7]. On the other hand, statin treatment accelerated the clearance of both LDE and native LDL [9].

Recently, we described an in vitro assay in whole plasma in which LDE was used as lipid donor to test the simultaneous transfer of lipids to HDL [10]. Lipid transfer is an essential step in the formation and metabolism of HDL, and allows the action of the lipoprotein in cholesterol esterification [11] and reverse cholesterol transport whereby cholesterol is transported to the liver and excreted in bile. Those are key processes for stabilization of the cholesterol pool in the organism.

In T1DM, strict glycemic control achieved by intensive insulin treatment can be beneficial for the prevention of premature cardiovascular events, the major complication of the disease [12]. In this approach, three or more daily insulin injections are administered, aiming to achieve glycemia as close as possible to normal levels. Insulin therapy can achieve lower LDL and higher HDL cholesterol levels [1] and may influence lipid transfers [4,13,14], composition of lipoproteins [13] and rate of LDL production [15].

An aspect that has not been sufficiently addressed is the status of lipid metabolism in T1DM patients under intensive insulin treatment in whom elevated glycemia persisted despite of the treatment. The aim of this study was to investigate this issue since control of dyslipidemia is key for the prevention of the diabetes-associated macrovascular disease, the major cause of morbidity and mortality of T1DM patients. The LDE plasma kinetic approach was used to test the LDL metabolic pathway and the transfer of lipids to HDL was evaluated by an in vitro assay [10]. Male T1DM patients under intensive insulin treatment but with high glycemic levels were compared with non-diabetic controls of similar age.

## Results

### Lipids and apolipoproteins

The levels of LDL tend to be lower in T1DM patients than in controls, although the difference did not attain statistical significance. Comparing the two groups, non-HDL cholesterol was lower, but HDL cholesterol and triglyceride levels were equal (Table 1). The levels of apo A1 and apo B were also equal in T1DM patients and controls (Table 1).

**Table 1:** Serum biochemical parameters of the Type 1 Diabetes Mellitus (T1DM) and control groups

	**T1DM group (n=15)**	**Control group (n=16)**	***P *value**
Fasting blood glucose (mg/dl)	192 ± 96	81 ± 4	<0.001
Glycated hemoglobin (%)	8.8 ± 1.5	5.2 ± 0.4	<0.001
Insulin sensitivity*	6.7 ± 2.0	9.8 ± 0.7	<0.001
Cholesterol (mg/dl)	155 ± 26	174 ± 31	0.09
LDL	83 ± 15	100 ± 29	0.08
HDL	55 ± 14	56 ± 9	0.88
Non-HDL	99 ± 17	117 ± 29	0.05
Triglycerides	80 ± 39	81 ± 40	0.94
Apolipoproteins (mg/dl)			
A1	127 ± 26	128 ± 17	0.91
B	67 ± 9	75 ± 23	0.26

Data are means ± SD. *Insulin sensitivity was estimated by glucose disposal rate (EGDR), when EGDR = 24.31-12.22(WHR)-3.29(hypertension)-0.57(A1c). WHR, waist-to-hip ratio.

### LDE plasma kinetics

**Figure 1:**
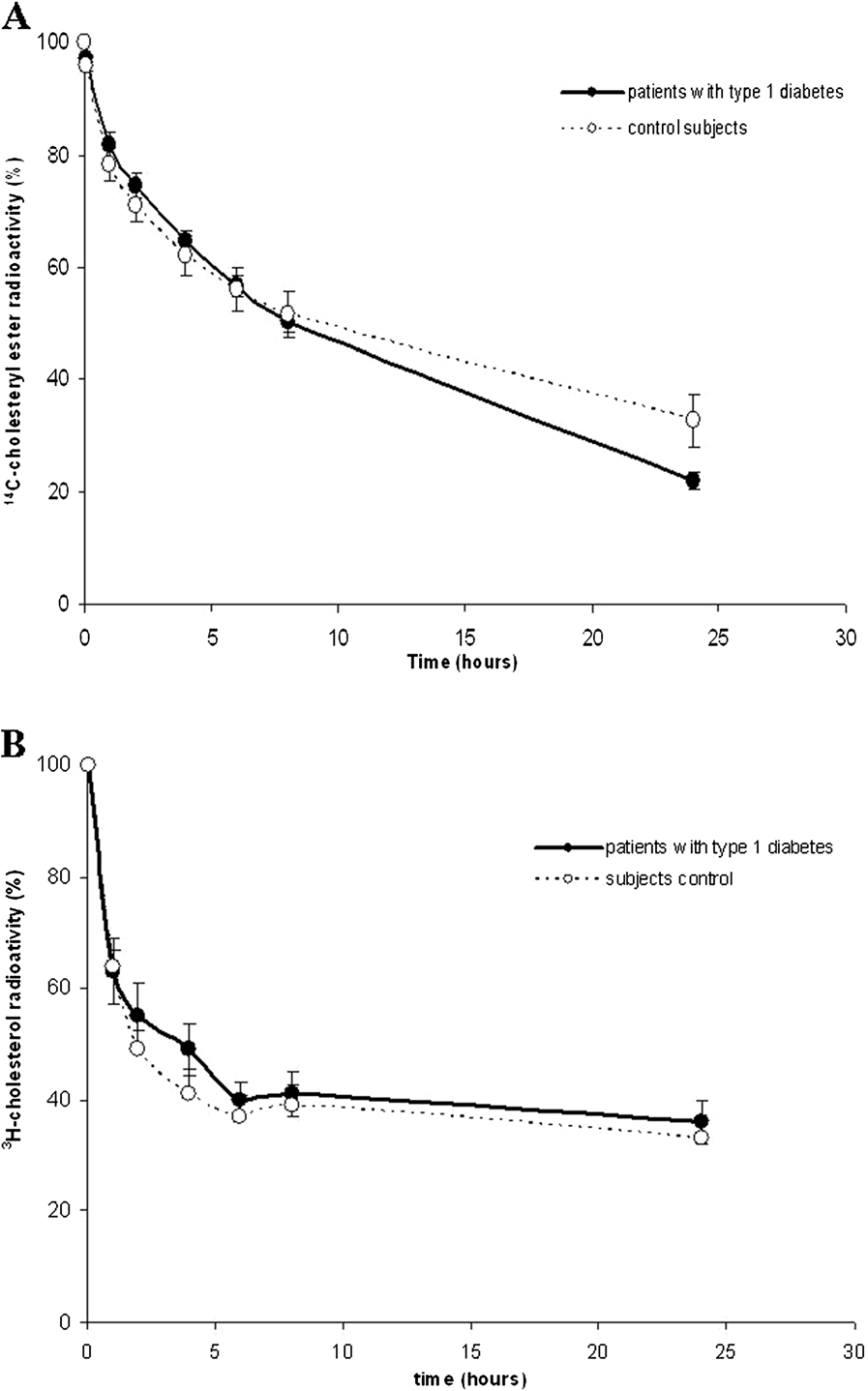
**Plasma decay curve of **^**14**^**C-cholesteryl ester (A) and **^**3**^**H-cholesterol (B) obtained from type 1 diabetes mellitus (black square) and control (white square) groups.** The doubly labeled nanoemulsion was intravenously injected in a bolus, and blood samples were drawn in pre-established intervals over 24 h for measurement of the radioactivity in a scintillation solution. Data are expressed as mean ± SD

As shown in Figure 1A, it is apparent that the decay curve of LDE ^14^C-cholesteryl esters obtained from the T1DM patients is faster than that of the control subjects. This is confirmed by the FCR of the LDE cholesteryl esters, that was greater in T1DM patients than in controls (0.059 ± 0.022 h^-1^ versus 0.039 ± 0.022 h^-1^, p=0.019) (Table 2). Greater FCR in T1DM was due to greater k2.0, since the other parameters of the compartmental analysis of the curves were not different in the two groups. In T1DM and controls, the FCR of LDE free cholesterol was equal.

**Table 2:** Fractional clearance rates and kinetic parameters of LDE radioactive lipid labels in the Type 1 Diabetes Mellitus (T1DM) and control groups

	**T1DM group (n=15)**	**Control group (n=16)**	***P *value**
FCR ^14^C-CE	0.059 ± 0.022	0.039 ± 0.022	0.019
k1.0 14C-CE	0.352 ± 0.278	0.502 ± 0.567	0.97
k2.0 14C-CE	0.049 ± 0.024	0.026 ± 0.017	0.006
k1,2 14C-CE	1.939 ± 1.464	1.436 ± 1.425	0.35
FCR ^3^H-FC	0.025 ± 0.024	0.028 ± 0.024	0.78
k1,0 3H-FC	0.911 ± 0.731	0.866 ± 0.651	0.86
k2,0 3H-FC	0.016 ± 0.021	0.011 ± 0.010	0.98
k1,2 3H-FC	0.999 ± 0.972	0.677 ± 0.815	0.19

Data are means ± SD. FCR ^14^C-CE, fractional clearance rate of ^14^C-cholesteryl ester. FCR ^3^H-CL, fractional clearance rate of ^3^H-free cholesterol. k_1,0_, constant that represents removal of lipid to the extravascular space. k_1,2_, constant that represents injected lipid which was converted into intravascular compartment*.* k_2,0_, constant that represents materials of intravascular compartment which is transferred to the extravascular space.

### Cholesterol esterification

The esterification rates of LDE free cholesterol after the injection into the T1DM patients were similar to that observed in the control subjects in all points of the decay curves (Table 3).

**Table 3:** **Esterification ratio (**^**3**^**H-cholesterol/**^**3**^**H-cholesteryl esters) in each point of the decay curves of labeled LDE injected into the Type 1 Diabetes Mellitus (T1DM) and control groups**

	**T1DM group (n=15)**	**Control group (n=16)**	***P *value**
0.08 h	29.4 ± 11.5	27.4 ± 6.9	0.60
1 h	40.3 ± 15.1	35.0 ± 10.3	0.35
2 h	48.8 ± 14.1	45.6 ± 8.9	0.55
4 h	51.1 ± 12.3	49.5 ± 11	0.83
8 h	61.7 ± 11.8	55.9 ± 9.2	0.23
24 h	65.7 ± 9.6	63.1 ± 9.1	0.51

Data are expressed as mean ± SD.

### Lipid transfers to HDL

The transfer of the radioactive free and esterified cholesterol, triglycerides and phospholipids from LDE to HDL was not different in T1DM and control subjects (Table 4).

**Table 4:** **Lipid transfer *in vitro *from LDE to HDL and HDL particle size of the Type 1 Diabetes Mellitus (T1DM) and control groups**

	**T1DM group (n=15)**	**Control group (n=16)**	***P *value**
Lipid transfers (%)			
Cholesteryl esters	2.7 ± 0,6	3.1 ± 0,8	0.09
Phospholipids	19.3 ± 3.7	20.7 ± 3.9	0.33
Tryglicerides	2.1 ± 0.8	2.3 ± 0.5	0.38
Free cholesterol	5.9 ± 1.7	6.0 ± 0.9	0.78
HDL particle size (nm)	10.4 ± 1.6	9.8 ± 1.2	0.20

Data are expressed as mean ± SD. The value indicated is the percentage of the radioactivity of each lipid component in the nanoemulsion that was transferred to the plasma HDL fraction after 1 hour incubation.

### Correlation analysis

In the correlation analysis performed between the data of glycemia, glycated hemoglobin, estimated glucose disposal rate and insulin dose per kilogram, on one hand, and the data of FCR of free and esterified cholesterol, cholesterol esterification rates and transfers to HDL of free and esterified cholesterol, triglycerides and phospholipids, on the other hand, no significant correlations were found.

## Discussion

In this study, although their having high glycemic levels, patients with T1DM under intensive insulin treatment showed a trend for lower LDL cholesterol, as well as faster removal of the LDE marker, ^14^C-cholesteryl ester as compared with the control subjects.

LDL cholesterol concentration in the plasma is determined by the balance between LDL production rates and the LDL removal from the plasma, which is largely dependent on the action of LDL receptors. In most clinical situations, slow LDL removal, rather than increased production rates, is the cause of hypercholesterolemia. Nonetheless, in a recent study [16], we have shown that the plasmatic removal of LDL, as monitored by LDE cholesteryl ester FCR, was faster in athletes than in sedentary subjects, although both groups had equal levels of LDL cholesterol. Those results suggest that the increase in LDL removal was compensated by increased LDL production. LDL turnover in the plasma should be more often renewed in athletes, and consequently the LDL peroxidation should be diminished [16]. In contrast in patients with familial hypercholesterolemia the plasma removal process of LDE is delayed [9]. Delay in LDL clearance makes room for increased lipoprotein peroxidation, uptake of oxidized LDL by macrophage scavenger receptors and subsequent formation of foam cells [17]. In the present study, in T1DM patients, the increased LDL removal, estimated by the LDE kinetics, resulted indeed in a trend for diminution of LDL, although not confirmed statistically. Non-HDL includes not only LDL but also other VLDL catabolic products such as intermediate density lipoprotein (IDL) and VLDL remnants that are also removed by LDL receptors or by LRP receptors [18]. Apo B concentration, that also marks the non-HDL lipoproteins, also tended to be lower in our T1DM patients. Insulin can act on LDL receptors by inducing receptor overexpression and increase in receptor activity [19]. Therefore, the fast removal of LDE, shown in the present study, was probably accounted for stimulation of the LDL receptors consequent to peripheral hyperinsulinemia in T1DM patients.

Insulin action on glycemia is exerted chiefly through glucose cellular uptake mediated by GLUT4 [20] and inhibition of glycogenolysis and gluconeogenesis [21]. The actions on lipoprotein metabolism are exerted mainly through increase in lipolysis of triglyceride-rich lipoproteins by stimulation of lipoprotein lipase [22], and inhibition of lipolysis of fats stored in the tissues by inhibition of hormone-sensitive lipase [23]. Apparently, the subcutaneous insulin dose scheme that was insufficient to optimize glycemia-related mechanisms of action had the ability to optimize the LDL pathway.

Depending on the route of insulin administration, the concentration of insulin in the peripheral circulation is different from that of the portal circulation, which conceivably has consequences on insulin availability and action and differences between subcutaneous and intraperitonial routes were documented [24]. Administration of excess exogenous insulin by subcutaneous route increases the catabolic rates of apo B LDL [25], similarly to our findings of increased LDE clearance in the T1DM patients. Accelerated LDL clearance diminishes the exposure of the lipoprotein to oxidation and other pro-atherogenic effects [25].

LDE ^3^H-free cholesterol removal from the plasma was similar in T1DM and controls, but the ^14^C-cholesteryl ester label is more reliable as tracer of LDE decay in the plasma than ^3^H-free cholesterol. In fact, ^3^H-free cholesterol kinetics was designed to evaluate the cholesterol esterification process in cases compared to controls. In the plasma, LDE also gains apo A1, the LCAT co-factor so that free cholesterol contained in LDE may suffer the esterification reaction [11]. Another possibility is that free cholesterol may partially shift from LDE to HDL, where it can also be esterified.

Cholesterol esterification may be related with CAD: in our previous study, we showed that in non-diabetic CAD patients cholesterol esterification was higher [26]. Nonetheless, in this study, in all points of the LDE decaying curves, cholesterol esterification did not statistically differ between T1DM and controls. Cholesterol esterification and LCAT activity have been poorly explored in T1DM. Chang et al. observed that the LCAT activity was higher in T1DM with higher glycemic levels than in those with lower glycemia [27], but comparisons with non-diabetic controls were not reported. Our data showing that LDE cholesterol esterification was equal in T1DM and controls may be helpful for future targeting the status of this reaction in T1DM with or without CAD.

T1DM patients treated with insulin also showed normal levels of fasting triglyceridemia. In diabetes, deficient function of insulin-dependent lipoprotein lipase may lead not only to hypertriglyceridemia but also to decreased HDL-cholesterol levels. This is the so-called “see-saw effect” triglycerides-HDL cholesterol consequent to increase in lipid exchanges between VLDL and HDL. In respect to HDL, not only the HDL-cholesterol and apo A1 levels were normal but also the metabolic step of lipid transfers to HDL was normal in the T1DM patients. Recently, it was shown that patients with documented coronary artery disease had alterations in lipid transfers to HDL such as decrease of free cholesterol transfers to the lipoprotein [28].

In a previous study performed in women with polycystic ovary syndrome [29], a correlation was found between insulin resistance, measured by the HOMA-IR index and that is associated with the syndrome, with the transfer of triglycerides to HDL. On the other hand, the action of both CETP and PLTP, that promote lipid transfer, was found to be altered in T1DM patients, and in another study CETP alterations were related with the presence of macrovascular disease [30]. Thus, lack of differences in lipid transfer in T1DM patients and controls may suggest that intensive insulin treatment could have contributed to the normal values in T1DM. Due to the importance of lipid transfer for HDL remodeling and function, this result, together with the finding of normal HDL-cholesterol and apo A1 levels adds up for the protection of intensive insulin treatment against macrovascular disease by maintenance of normal plasma lipid metabolism pathways.

Native LDL is recognized by LDL receptors by means of apo B, the only apo present in this lipoprotein. Recognition of LDE by receptors through apo E, which has greater affinity for the receptors results in removal of LDE considerably faster than the native lipoprotein[8], which facilitates the execution of the experimental protocols and the comfort of the study subjects by shortening the blood sampling period. In many studies, LDE kinetics was accelerated or slowed as previously reported for the native lipoprotein [31-33]. Nonetheless, as a limitation of those studies, the differences in protein recognition by the receptors and the compositional differences between LDE and the native lipoprotein are to be taken into account in the interpretation of the LDE-generated data for extrapolation to LDL metabolism.

It is worthwhile to point out that despite the beneficial effect on lipoprotein metabolism, as documented in this study, patients under intensive insulin therapy have reportedly higher cardiovascular mortality risk [34,35]. This may be ascribed to the short follow-up periods of the insulin-treated patients, since atherosclerosis is a long-run process. In fact, in studies with 17 and 20 year follow-up, intensive insulin therapy achieved reduction of events in both T1DM and type 2 diabetes mellitus; patients were younger and had no manifestations of cardiovascular disease when they begun the treatment [36,37].

In conclusion, although intensive insulin treatment was not sufficient to normalize glycemia, no abnormalities were found in the aspects of lipid metabolism examined here; the LDE clearance was even increased. Presumably, this dichotomy could have resulted from different thresholds of insulin levels to optimize glucose, on one hand, and plasma lipid metabolism, on the other. Insulin bioavailability after subcutaneous administration was also conceivably key for this outcome. The fact that under insulin treatment optimal lipid parameters can be attained independently of the glycemic control could be fundamental because of the importance of dyslipidemias in the development of diabetic macrovascular disease.

## Methods

### Study subjects

Fifteen male young patients with T1DM and 16 non-diabetic healthy male subjects studied at the Outpatient Diabetes Clinics of the Medical School Hospital of the University of São Paulo (São Paulo, Brazil).

None of subjects had history of coronary artery disease, dislipidemia, thyroid, gastrointestinal, inflammatory, kidney or liver disease or history of excessive alcohol drinking. T1DM patients were free from apparent micro and macrovascular diabetic complications. None of the participants were using lipid lowering drugs. Table 5 shows the insulin treatment scheme of the patients. As indicated in Table 1, their glycated hemoglobin was greatly elevated.

**Table 5:** Physical characteristics and current medications of the Type 1 Diabetes Mellitus (T1DM) and control groups

	**T1DM group (n=15)**	**Control group (n=16)**	***P *value**
Age (years)	26.4 ± 7.0	28.4 ± 6.0	0.40
Age of diabetes diagnosis (years)	12.2 ± 7.2	-	-
Duration of diabetes (years)	15.7 ± 7.9	-	-
Body mass index (Kg/m^2^)	23.2 ± 2.3	25.7 ± 3.6	0.04
Waist circumference (cm)	84.3 ± 8.3	90.5 ± 9.4	0.09
Waist-to-hip ratio	0.83 ± 0.04	0.82 ± 0.05	0.80
Current smoking (%)	0	25	0.09
Arterial hypertension (%)	2	0	0.23
Insulin therapy schemes			
Insulin dose (U · day^– 1^)	48.7 ± 13.8	-	-
Insulin dose (U · kg^– 1^ · day^– 1^)	0.69 ± 0.15	-	-
Insulin injections/day	3.53 ± 0.748	-	-
Insulin correction bolus (%)	60	-	-
Carbohydrate counting (%)	40	-	-

Data are means ± SD.

All participants gave written informed consent. Studies were approved by the Ethics Committee of the Medical School Hospital of University of São Paulo.

### Laboratory analysis

After an overnight fast, blood samples were taken and total cholesterol, HDL cholesterol, and triglyceride levels were measured using enzymatic colorimetric methods. LDL cholesterol was calculated by direct method (kinetic automatized). Plasma apoA-I and apoB were assayed by turbidimetry (Roche/ Hitachi – Roche Diagnostics, Mannheim, Germany).

A1C was measured by HPLC (National Glyco Hemoglobin Standardization Program - NGSP-EUA, considering normal range 4.1 a 6%). Plasma glucose concentration was measured by a hexokinase method. Insulin sensitivity was assessed by estimated glucose disposal rate [38], calculated according to the formula: estimated glucose disposal rate (EGDR) = 24.31 - 12.22*(WHR) - 3.29*(hypertension) - 0.57*(A1C). The equation was derived from hyperinsulinemic-euglycemic clamps performed in 24 participants with type 1 diabetes in the Pittsburgh Epidemiology of Diabetes Complications (EDC) Study [38]. Original EGDR formula utilizes A. To convert A1C in A1, the equation HbA1= 1.18 x A1C + 1.67 was used [39].

### LDE preparation

LDE was prepared from a lipid mixture composed of 40 mg cholesteryloleate, 20 mg egg phosphaditylcholine, 1 mg triolein, and 0.5 mg cholesterol. ^14^C-cholesteryl oleate and ^3^H-cholesterol purchased from Amersham International (Amersham, UK) were added to the mixture. The lipid mixture was then submitted to an ultrasonic irradiation in aqueous media, dried through nitrogen flux, and purified by a procedure of a two-step ultracentrifugation procedure, as described previously [5]. LDE was dialyzed againsta saline solution and sterilized by passage through 0.22-μm filter.

The entire nanoemulsion preparation procedure was performed in a laminar flux. All glassware used in this study was made pyrogen-free by exposure to dried steam at 180°C for 2 hours and sterilized by wet steam at 120°C for 30 min. All plastic materials were sterilized by ultraviolet light exposition. The risk due to the nanoemulsion injection is considered minimal and related to the consequences inherent to venous puncture. The risk of infection is also minimum, since preparations were tested for sterility and absence of pyrogen. Furthermore, the injected nanoemulsion volume was extremely small, which reduces the chance of pyrogenic reaction.

### LDE plasma kinetics

The test began at approximately 8:00 AM with all participants fasting for a 12 h period. One hundred microliters of LDE containing 37 kBq ^14^C-labeled cholesteryl ester and a total of 1 mg cholesteryl ester was intravenously injected in a bolus. Plasma samples were collected during 24 h, in intervals of 5 min and 1, 2, 4, 6, 8, and 24 h after injection. During the first 8 h of the sample collection, all the participants were resting in the laboratory test room and remained seated. They were then allowed to return home and come back to the laboratory the next day to collect the last (24 h) blood sample. They were oriented to rest at home. After the first blood collection, the subjects were oriented to eat low-fat meals, as vegetables, fruits, and fruit juices after the first blood collection.

Radioactivity in aliquots of 1.0 ml of plasma was quantified in a scintillation solution (Packard Bioscience, Groningen, Netherlands) using a liquid scintillation analyzer (Packard 1600 TR Model liquid scintillation analyser, Palo Alto, CA).

### Estimation of FCR of the radioisotopes

FCR of the LDE ^14^C-cholesteryl ester, as calculated as described previously [34] by compartimental analysis using a computational software (ANACOMP by C.H. Mesquita, São Paulo, Brazil) [40,41].

### Esterification of the emulsion ^3^H-cholesterol

Using 1.5 ml blood plasma in each point of timed colleted blood during 24 h, lipids were extracted with chloroform-methanol–water (2:1:1, v/v/v) overnight at 4°C. The organic phase was transferred to a test tube and dried under nitrogen flux. Lipids were resuspended in 5 ml of the Folch’s solution (chlorofom-methanol, 2:1, v/v) and transferred for test tubes. Next, the tubes were dried under nitrogen flux, ressuspended with 300 μl of Folch’s solution and submitted to separation by thin layer chromatography (TLC) (silica-gel 60H, 0.5 mm thickness), with a solvent system containing n-hexane: Ethylic ether: acetic acid (70:30:1, v/v/v). The bands that corresponded to cholesteryl ester and to free cholesterol, after being developed by metallic iodine vapors, were separated and transferred to counting vials containing 7.0 ml of scintillation solution.

An esterification ratio was calculated to determine the proportion of unesterified to esterified cholesterol at each experimental point (5 min and 1,2, 4, 6, 8 and 24 h) of the radioactivity decay curve by dividing the ^3^H radioactivity measured after lipid extraction at the unesterified cholesterol TLC band by that of the esterified cholesterol band measured.

### Lipid transfer from LDE to HDL

Two sets of LDE preparations, one labeled with ^3^H-cholesteryl oleate and ^14^C-phosphatidylcholine and the other with ^3^H-triolein and ^14^C-cholesterol, were separately incubated with whole plasma in a shaking bath for 60 min at 37°C and after chemical precipitation of apoB containing lipoproteins and LDE, the supernatant containing HDL was counted for radioactivity, according to the method described previously [10]. The transferred lipids from LDE to HDL was expressed as percent (%) of the radioactivity in the incubate measured in the supernatant.

### Radiological protection

The radioactive dose used in the intravenously injected labeledlipid experiments with the patients was in strict accordance with the International Commission on Radiological Protection (ICRP), as described in a previous study [42]. The equivalent dose induced by the injected radioactivity dose was 0.03 mSv, well below the permitted 50.0 mSv maximum dose.

### Statistical analyses

Data are presented as mean ± SD. The Kolmogorov-Smirnov test was applied to verify the data distribution.The Student *t*-test was used for normally distributed data, and the Mann–Whitney test for those with non-Gaussian distribution. To determinate relationship between glycemia, A1C, insulin sensitivity and insulin therapy with kinetic and HDL assays Pearson's correlation coefficients and Spearman's correlation coefficients were calculated according data distribution. SSPS version 13 software was used for the analysis.

For all comparisons and correlations, p<0.05 were considered statistically significant.

## Competing interests

The authors declare that they have no competing interests.

## Authors’ contributions

ACRF selected the patients, performed the experiments and data analysis, and wrote the manuscript. GSFF performed the experiments and data analysis. FRF contributed to discussion and wrote the manuscript. BLW designed the clinical protocol, and contributed to discussion. RCM conceived of the study, analyzed the results, and wrote the manuscript. All authors read and approved the final manuscript.
